# An Observation-Based Dataset of Global Sub-Daily Precipitation Indices (GSDR-I)

**DOI:** 10.1038/s41597-023-02238-4

**Published:** 2023-06-22

**Authors:** David Pritchard, Elizabeth Lewis, Stephen Blenkinsop, Luis Patino Velasquez, Anna Whitford, Hayley J. Fowler

**Affiliations:** Newcastle University, Newcastle upon Tyne, UK

**Keywords:** Atmospheric science, Climate change

## Abstract

Precipitation indices based on daily gauge observations are well established, openly available and widely used to detect and understand climate change. However, in many areas of climate science and risk management, it has become increasingly important to understand precipitation characteristics, variability and extremes at shorter (sub-daily) durations. Yet, no unified dataset of sub-daily indices has previously been available, due in large part to the lesser availability of suitable observations. Following extensive efforts in data collection and quality control, this study presents a new global dataset of sub-daily precipitation indices calculated from a unique database of 18,591 gauge time series. Developed together with prospective users, the indices describe sub-daily precipitation variability and extremes in terms of intensity, duration and frequency properties. The indices are published for each gauge where possible, alongside a gridded data product based on all gauges. The dataset will be useful in many fields concerned with variability and extremes in the climate system, as well as in climate model evaluation and management of floods and other risks.

## Background & Summary

Datasets providing time series of climate indices are essential for understanding climate variability and change^1^. Focusing on precipitation and temperature extremes, the WMO/WCRP/JCOMM Expert Team on Climate Change Detection and Indices (ETCCDI) developed a set of indices around two decades ago that is still in wide use today in global climate change detection studies^2,3^ and climate model evaluations^4^. A key advance made by the ETCCDI was to standardise both the definitions and the software used in index calculation. These steps enabled direct comparison between analyses and wider sharing of vital observed climate information, as some data holders are able to derive and share indices more readily than raw climate data. A succession of open, station-based and gridded observational datasets has followed from the introduction of the ETCCDI indices, including HadEX^5^, HadEX2^6^, HadEX3^7^ and GHCNDEX^8^.

As the ETCCDI indices are calculated from daily data, they do not provide any information on variability, changes or extremes of precipitation at sub-daily timescales. Indeed, sub-daily precipitation data from most countries around the world are generally not openly available. Yet, intense precipitation at short durations can have very large impacts, including flash flooding^9^, landslides^10^ and soil erosion^11^. Available observations and high-resolution (convection-permitting) climate modelling indicate that these high-impact extremes will intensify in a warmer and moister atmosphere^12–16^, which may lead to increases in the associated risks to life and higher economic costs. At large scales some regions may experience sub-daily precipitation intensity increases greater than the ~7% K^−1^ implied by the Clausius-Clapeyron relation^17,18^, although the interplay between cloud processes, local dynamics and large-scale circulation exerts a complex modulating influence on the thermodynamic scaling of precipitation extremes with warming^19,20^.

It is therefore critical to increase the availability of open sub-daily precipitation information to support both climate research and risk management. As such, this study presents a new global dataset of sub-daily precipitation indices (GSDR-I) calculated from a unique database of gauge observations. This work was conducted in the INTENSE project (‘INTElligent use of climate models for adaptatioN to non-Stationary hydrological Extremes’)^21^. INTENSE represented a major international effort to improve understanding of changing sub-daily precipitation extremes around the world. The project led the global research effort in this area within the Global Energy and Water Exchanges Hydroclimatology Panel Cross-Cutting project on sub-daily precipitation as part of the World Climate Research Programme.

**Fig. 1:**
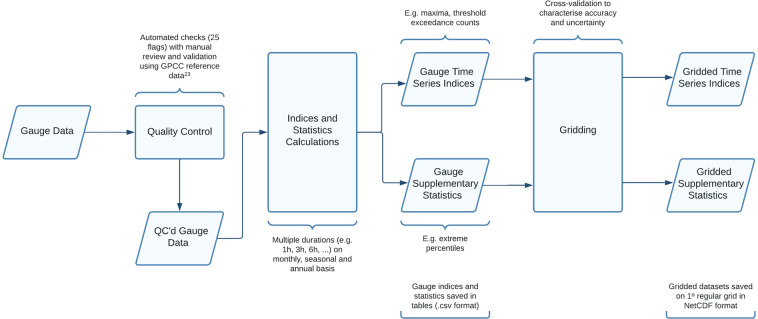
Summary of the data flow, workflow and methods used to develop the GSDR-I sub-daily precipitation indices dataset.

Figure 1 outlines the approach taken to construct GSDR-I. The first step was to collate and quality control as much global sub-daily precipitation data as possible. This culminated in the Global Sub-Daily Rainfall (GSDR) dataset^22^. Building on previous work, an open-source automated quality control procedure (GSDR-QC) was developed, evaluated and applied to the GSDR dataset^23^. In parallel, the INTENSE project facilitated discussions with international experts and data users to help define a set of sub-daily precipitation indices for GSDR-I. Python code was then developed to calculate these indices for one or more gauges. This software was shared with partners who wished to calculate the indices themselves and contribute them to GSDR-I. Finally, the indices calculated from gauge records were gridded, which enabled the incorporation of gauges whose indices cannot be released directly due to licensing restrictions. The gridded indices form a data product comparable to the ETCCDI-related indices datasets (HadEX3, GHCNDEX) but focused on sub-daily timescales.

The resulting indices dataset will be useful in many areas of research and applied risk management. It will provide climate researchers with the most comprehensive observation-based dataset on the climatology and variability of sub-daily precipitation available. Climate modellers will be able to use the dataset to help evaluate and improve models operating at a range of scales, from global to regional and local (including convection-permitting models). In addition, through the new indices dataset, engineers and risk managers will have access to unprecedented open information on observed short-duration precipitation extremes relevant for assessing hazards and designing resilience measures. For example, the indices describing monthly, seasonal and annual maxima will be directly applicable in extreme value analysis and the construction of intensity-duration-frequency curves, which are used to design drainage systems and flood resilience measures. Further updates to the indices dataset over time will also ultimately enhance its role in climate change detection.

## Methods

### Gauge observations

The INTENSE project undertook a major effort to collate sub-daily precipitation gauge records from around the world. This activity culminated in the construction of the GSDR hourly precipitation dataset^22^, which provides the majority of the data underpinning the GSDR-I sub-daily indices calculations. National meteorological and hydrological services provided most of the data for the GSDR, although other environmental agencies and researchers also contributed substantially. Gauges from the Integrated Surface Database (ISD)^24^ were also included if they were not present in data obtained from national hydrometeorological services. Compared with the initial version described by Lewis *et al*.^22^, the GSDR now contains data for 18,591 gauges, due to the addition of hourly records for Brazil (765 gauges; https://portal.inmet.gov.br/), Canada (1705 gauges; https://climate.weather.gc.ca/), India (65 gauges; https://mausam.imd.gov.in/), Ireland (36 gauges; https://www.met.ie/climate/available-data/historical-data) and New Zealand (453 gauges; https://cliflo.niwa.co.nz/).

**Fig. 2:**
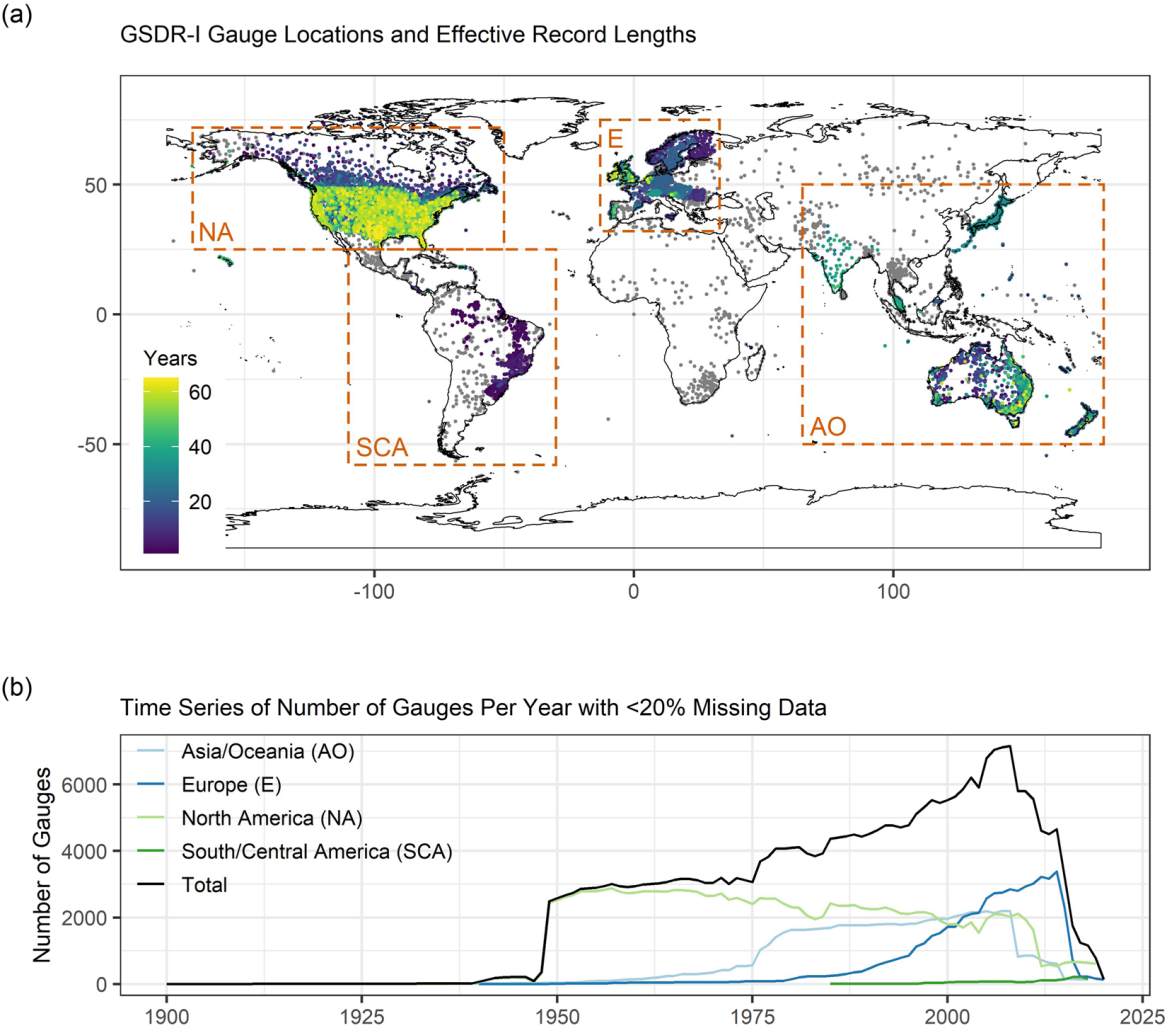
Summary of hourly gauge data records used in indices calculations. The spatial distribution of gauges is shown in (**a**), with effective record lengths defined as the product of the total record length and the record completeness (indicated via colour scale). Effective record length is only given where it exceeds 1 year (gauges with shorter records are shown in grey). Over-plotting occurs where gauge density is high (and longer effective record lengths are plotted on top of shorter ones). Annual time series of the number of gauges with <20% missing data are shown in (**b**) for four regions containing gauges with effective record lengths of >1 year. Region definitions are provided in (**a**). Both panels reflect data availability after quality control was applied to the time series.

Figure 2a shows the effective record lengths (i.e. equivalent number of years of record if missing data are removed) of gauges used to build the GSDR-I dataset. Only hourly gauge records with effective lengths exceeding one year were used in the indices calculations. Figure 2 indicates that data coverage is highly variable, with long records available mainly in parts of Europe, Asia, Australasia and North America. The number of stations available in these regions varies over time (Fig. 2b), which partly reflects changing measurement networks but also difficulties in obtaining up-to-date records globally. Peak gauge availability globally in GSDR-I is found in 2008, when 7,150 relatively complete gauges (<20% missing) contribute to the dataset simultaneously. Data is very limited in much of South America and Africa, as well as parts of Asia and the Middle East. Nevertheless, with 12,104 relatively complete (<20% missing) gauge records of more than one year in length, the GSDR is the most comprehensive dataset of global hourly precipitation observations and hence the basis for GSDR-I.

It should also be noted here that indices calculations for additional gauges (not held in GSDR) were performed and shared by meteorological services from all member countries of the PannEX initiative^25^. PannEX is a GEWEX Regional Hydroclimatological Project (RHP) focusing on the hydroclimatology of the Pannonian basin through collaboration by Austria, Croatia, Czech Republic, Hungary, Romania, Serbia, Slovakia and Slovenia. PannEX contributed 264 of the total 18,591 gauges in the GSDR-I indices dataset.

### Quality control

Errors in precipitation observations arise from various sources, including equipment malfunctions (e.g. gauge blockages) and data/metadata transmission or processing errors (e.g. missing values infilled with zeros, implausible values, etc.). For sub-daily precipitation, some specific additional issues tend to recur in data series, such as entry of daily accumulations at single hours or by uniform disaggregation. However, only some of the data collected for GSDR had associated time series of quality flags. Therefore, given the range of potential quality issues and the volume of data in GSDR, the INTENSE project developed a largely automated quality control procedure to identify and remove suspicious data. This procedure (GSDR-QC^23^) builds on previous work on automated checking of daily^24^ and sub-daily^26,27^ precipitation data to underpin an open-source Python package (*intense*) for community use.

The GSDR-QC procedure comprises several steps, with full details and evaluation given by Lewis *et al*.^23^. First, station metadata checks identify and exclude implausible locations and duplicated records. Second, a set of 25 time series checks are used to flag suspicious data values or periods. These checks cover a wide range of possible errors, including implausible extreme values, entry of daily accumulations into hourly series, suspicious streaks of repeated values, dubiously long dry periods relative to local climate, record inhomogeneity, excessive intermittency, and inconsistency in relation to neighbouring reference gauges, amongst others. These checks draw on consistency with well verified auxiliary data in the form of HadEX2 and GHCNDEX gridded ETCCDI daily indices, as well as with the high quality daily and monthly precipitation observations curated by the Global Precipitation Climatology Centre (GPCC)^28^.

Values and periods flagged as very suspicious in the second step are removed from the time series based on a set of 11 rules explained in Lewis *et al*.^23^. The rules focus on excluding the following errors from the time series: suspected daily and monthly accumulations; long streaks of repeated values; implausible extremes relative to known records, daily extreme totals and neighbouring gauges (after applying a safety margin); and long dry periods not corroborated by ETCCDI datasets or neighbouring gauges. These rules combine multiple time series checks to enhance their robustness. The performance of the GSDR-QC procedure “rule base” is discussed in the Technical Validation and Usage Notes sections below. A final manual review then identifies and removes gauges whose overall records are of particularly poor quality, based on examination of the GSDR-QC summary information. After this review for both GSDR gauges and those gauges contributed by PannEX, 18,591 gauges remained available for use in construction of the GSDR-I indices dataset.

### Definitions of indices

Two objectives guided the development of the indices and statistics included in GSDR-I. The first objective was compatibility with the ETCCDI indices as far as possible. This principle will enable users to compare GSDR-I indices with longer duration (daily and longer) precipitation indices, while also making the new sub-daily indices readily understandable. The second objective was to ensure user/stakeholder participation in the design of new indices. To this end, the INTENSE project held a workshop in September 2016 involving climate scientists, modellers and flood risk specialists to identify and define appropriate, useful indices and statistics. This process resulted in the GSDR-I indices summarised in Table 1.

**Table 1:** Overview of time series indices in GSDR-I.

Group	Name	Equation	Description	Units
Intensity	Rx*H*hr	9	Simple monthly, seasonal or annual maxima of *H*-hour (1-, 3-, 6-, 12- or 24-hour) precipitation series (using a sliding window to identify maxima when *H* > 1)	mm
General	Rx1hrP	10	Percentage contribution of Rx1hr (1-hour maximum) to the total precipitation of the corresponding calendar day (00:00-00:00 local time)	%
General	R*Q*pw*H*hrP	8	Percentage contribution to monthly, seasonal or annual totals from *H*-hour intervals exceeding the *Q*th (95^th^ or 99th) wet-hour (*w*) percentiles (see R*Q*pw*H*hr in Table 2)	%
Frequency	R*H*hr*T*mm	6	Count of hours (or multi-hour intervals if *H* is greater than 1) greater than 10, 20, 30 or 50 mm thresholds (*T*)	count
Duration	NWH	1	Total number of wet hours	hours
Duration	MeLWS	5	Mean length of wet spell	hours
Duration	MxLWS	4	Maximum length of wet spell	hours
Intensity	SPII*H*hr	7	Simple precipitation intensity index, defined as mean precipitation in wet hours (or multi-hour intervals if *H* is greater than 1)	mm
General	RTot	3	Total precipitation	mm

In the index names, *H* represents the accumulation interval (e.g.1-hour, 3-hour) of the precipitation series used in index calculation. A threshold of 0.1 mm is used to identify wet hours (or multi-hour intervals) where relevant. A wet spell is defined by consecutive wet hours and is terminated by one or more dry hours.

Table 1 shows that the GSDR-I time series indices build on common and typically simple concepts, such as maxima and counts over thresholds, which generally have counterparts in the ETCCDI indices. For example, GSDR-I contains an Rx1hr index defined as a time series of 1-hour precipitation maxima, whereas the ETCCDI has Rx1day to describe a 1-day maxima series. Similarly, the GSDR-I dataset (like the ETCCDI-based datasets) also incorporates explicit frequency-related indices to help users quantify exceedances of fixed thresholds, which are often relevant indicators for physical risk managers. Other indices provide information on the contribution of heavy precipitation to total amounts on scales ranging from daily to annual, as well as wet spell durations and mean/total precipitation.

All of the time series indices in Table 1 are calculated on a monthly, seasonal and annual basis to provide flexibility to the data users. Seasons are defined as December to February (DJF), March to May (MAM), June to August (JJA), and September to November (SON). A threshold of 0.1 mm is used to distinguish wet hours (or multi-hour intervals) in the relevant calculations. All of the indices are calculated using a 1-hour accumulation interval (i.e. using hourly gauge time series) and, with the exceptions of Rx1hrP, RTot and the duration-related indices (NWH, MeLWS and MxLWS), most indices are also calculated using 3-, 6-, 12- and 24-hour accumulation intervals (i.e. after aggregating the hourly (1-hour) gauge time series) to facilitate as wide a range of user applications as possible. For most indices, aggregations of the 1-hour series use fixed windows beginning at 00:00 local time, e.g. 00:00–03:00, 03:00–06:00, etc. However, maxima (RxHhr) are identified using a sliding window, which provides a more accurate approximation.

Following the ETCCDI indices, we also provide formal definitions of the GSDR-I time series indices in Table 1. Let *X*_*ij*_ be the precipitation total for the *i*-th successive hour in the *j*-th period (month, season or year) of an hourly precipitation time series, *X*. Let *n*_*j*_ denote the number of hours (wet and dry) in period, *j*. Using Iverson bracket notation, the number of wet hours in period *j* can be obtained from

1 $$NW{H}_{j}={\sum }_{i=1}^{{n}_{j}}\left[{X}_{ij}\ge 0.1\right]$$

where [*X*_*ij*_ ≥ 0.1] is an indicator function mapping *X*_*ij*_ to one if it equals or exceeds 0.1 mm and to zero otherwise, i.e.

2 $$\left[{X}_{ij}\ge 0.1\right]=\left\{\begin{array}{c}1,{X}_{ij}\ge 0.1\\ 0,{X}_{ij} < 0.1\end{array}\right.$$

The total precipitation in wet hours for period *j* can be identified from

3 $$RTo{t}_{j}={\sum }_{i=1}^{{n}_{j}}{X}_{ij}\left[{X}_{ij}\ge 0.1\right]$$

where *X*_*ij*_ [*X*_*ij*_ ≥ 0.1] indicates that *X*_*ij*_ is only included in the summation if it is greater than or equal to 0.1 mm.

If *LWS*_*rj*_ is the length (in hours) of continuous wet spell *r* (in which each hour equals or exceeds 0.1 mm) in period *j*, then the maximum wet spell length of all *w*_*j*_ wet spells in period *j* can be written as

4 $$MxLW{S}_{j}=\left\{\begin{array}{cc}\mathop{\max }\limits_{r=1,\ldots ,{w}_{j}}LW{S}_{rj}, & {w}_{j} > 0\\ 0, & {w}_{j}=0\end{array}\right.$$

and the mean wet spell length is calculated from

5 $$MeLW{S}_{j}=\left\{\begin{array}{cc}\frac{{\sum }_{r=1}^{{w}_{j}}LW{S}_{rj}}{{w}_{j}}, & {w}_{j} > 0\\ 0, & {w}_{j}=0\end{array}\right.$$

Now let *H* ∈ {1, 3, 6, 12, 24} be an accumulation interval in hours and *Y*_*kj*_ be the precipitation total for the *k*-th successive (non-overlapping) *H*-hour interval in the *j*-th period (month, season or year) – i.e. after aggregation (summation) of the original hourly series (*X*) using fixed *H*-hour intervals starting at midnight local time to obtain series *Y* (which thus has a *H*-hour interval or time step). There are *m*_*j*_ successive *H*-hour intervals in *Y*_*j*_. For a threshold *T* ∈ {10, 20, 30, 50} (in mm) and using the indicator function [*Y*_*kj*_ ≥ *T*] to map *Y*_*kj*_ to one if it is greater than or equal to *T* (otherwise it is mapped to zero), we can identify counts of exceedances from

6 $$RHhrTm{m}_{j}={\sum }_{k=1}^{{m}_{j}}\left[{Y}_{kj}\ge T\right]$$

In a similar way, mean precipitation intensity in wet *H*-hour intervals is defined as

7 $$SPIIHh{r}_{j}=\begin{array}{cc}\frac{RTo{t}_{j}}{{\sum }_{k=1}^{{m}_{j}}\left[{Y}_{kj}\ge 0.1\right]}, & RTo{t}_{j}\ge 0.1\\ 0, & RTo{t}_{j} < 0.1\end{array}$$

Then let *Y*_*Q*_ be a threshold defined as the *Q*-th percentile (with *Q*∈{95, 99}) of the precipitation distribution calculated from wet *H*-hour intervals (≥0.1 mm) using all months, seasons or years in the time series that share the same the month, season or year of period *j* (e.g. all Januarys). The contribution of *H*-hour intervals exceeding *Y*_*Q*_ to the total precipitation in wet intervals for period *j* can be calculated as

8 $$RQpwHhr{P}_{j}=\left\{\begin{array}{cc}\frac{{\sum }_{k=1}^{{m}_{j}}{Y}_{kj}\left[{Y}_{kj}\ge {Y}_{Q}\right]}{RTo{t}_{j}}\times 100 \% , & RTo{t}_{j}\ge 0.1\\ 0, & RTo{t}_{j} < 0.1\end{array}\right.$$

where *Y*_*kj*_ [*Y*_*kj*_ ≥ *Y*_*Q*_] indicates that *Y*_*kj*_ is only included in the summation if it is greater than or equal to *Y*_*Q*_.

Finally, for the maxima-related indices, we take *H*∈{1, 3, 6, 12, 24} again as an accumulation interval in hours and let *Z*_*hj*_ be the precipitation total for the *H*-hour interval ending at hour *h* in the *j*-th period (month, season or year) of an hourly time series *X*_*j*_. *Z*_*j*_ is thus an hourly time series based on a sliding window aggregation, in which the value at each hour *h* is a sum of itself and the *H*-1 preceding hours. With *n*_*j*_ as the total number of hours (wet and dry) in period *j*, the *H*-hour maximum can be determined from the sliding window series by

9 $$RxHh{r}_{j}=\mathop{\max }\limits_{h=H,H+1,\ldots ,{n}_{j}}{Z}_{hj}$$

If *D*_*j*_ is the 24-hour precipitation total on the calendar day (00:00 – 00:00 local time) in which the 1-hour maximum precipitation *Rx*1*hr*_*j*_ occurred, then

10 $$Rx1hr{P}_{j}=\left\{\begin{array}{cc}\frac{Rx1h{r}_{j}}{{D}_{j}}\times 100 \% , & Rx1h{r}_{j} > 0\\ 0, & Rx1h{r}_{j}=0\end{array}\right.$$

If more than one hour in period *j* has the value *Rx*1*hr*_*j*_ then one of those hours is chosen at random to identify *D*_*j*_.

### Definitions of supplementary statistics

In addition to the time series indices, the GSDR-I dataset also contains a small number of supplementary statistics calculated as single values based on full record periods (e.g. percentiles), rather than as time series. Detailed in Table 2, these statistics describe aspects of short-duration precipitation climatology – specifically extreme precipitation percentiles and the diurnal cycle/profile of precipitation. The supplementary statistics are provided because they contain useful information for interpreting the time series indices (i.e. percentiles) or for particular applications, such as climate model evaluation, while requiring longer, climatology-length record periods for their definition.

**Table 2:** Overview of supplementary statistics.

Group	Name	Description	Units
Percentiles	R*Q*pw*H*hr	*Q*-th (95th, 99th) percentile of the precipitation distribution for wet *H*-hour intervals (*w*) based on a 0.1 mm threshold	mm
Percentiles	R*Q*pa*H*hr	As above but for the all-hour (*a*) distribution (dry and wet)	mm
Diurnal Cycle	DiCyc1mm	Mean precipitation for each hour of the day on wet days exceeding a 1 mm threshold	mm

In the names, *H* represents the accumulation interval (in hours) of the precipitation series used in statistic calculations. Diurnal cycle indices are calculated using a 1-hour accumulation interval, while percentiles are calculated based on 1-, 3-, 6-, 12- and 24-hour accumulation intervals too.

Table 2 documents the GSDR-I supplementary statistics, each of which comprise climatological values rather than time series. The supplementary statistics include all-hour and wet-hour percentiles (i.e. as opposed to the time series indices of percentage contributions above percentile thresholds – see Table 1). Providing both percentile variants enables flexibility for different applications, while ensuring that influences of frequency variability can be considered in analyses of intensity^29^. Wet hours (or multi-hour intervals) are again defined using a 0.1 mm threshold. The diurnal cycle is also calculated on a climatological basis, in acknowledgement of the fact that some climate regions experience few wet days, at least seasonally. The full diurnal cycle (i.e. mean hourly precipitation by hour of day) is thus calculated as a climatology using wet days throughout the available record period. Wet days have been defined using a 1 mm threshold (DiCyc1mm). Days are defined as calendar days, i.e. 00:00 to 00:00 local time. The number of wet days in a period is recorded as metadata (see Data Records section). The supplementary statistics are calculated on a monthly, seasonal and annual basis (e.g. pooling all Januarys together).

### Calculation details

Several additional details are important in the practical calculation of the GSDR-I indices and statistics for individual gauges. Firstly, for all of the supplementary statistics in Table 2, calculations use full available record periods, rather than a common reference period (e.g. 1981–2010). This approach is taken because of the highly variable length of record periods within GSDR, as well as the substantial differences in start and end years of records. It is very difficult to define a common reference period across the GSDR dataset without eliminating a substantial amount of data or having potentially low record completeness. Sub-daily gauge records are typically much shorter than daily records, such that it is important to retain as much information as possible. Therefore, to help users understand the length and timing of the records used, the metadata includes the record period of each gauge and its completeness.

It was also necessary to handle missing data in the calculations. For the time series indices, percentage completeness is recorded alongside the index value for each month, season or year calculated. The user can then filter time series indices using a data completeness threshold of their choice. For the supplementary statistics, missing data was accounted for by performing two sets of calculations: (1) using all available data, regardless of completeness over the record period (i.e. a completeness threshold of 0%), and (2) performing the calculation only on those months, seasons or years with more than 80% completeness for the period required. The number of data points available for calculation is given as metadata in both cases. This approach gives some flexibility to the dataset user, who can examine the impact of restricting calculations to relatively complete periods on their results.

The GSDR-I index calculations were implemented in Python (see Code Availability section below). Similar to the software for the ETCCDI indices, the code enables consistent calculations by different users. This is a very useful property in cases where national meteorological services are unable to share full data records. Indeed, sharing the Python package enabled the contribution of indices for 264 gauges not held in the underlying GSDR dataset by the PannEX initiative^25^, as outlined above.

### Gridding: Angular distance weighting

Gridding the GSDR-I gauge-based indices represents a way to include the maximum amount of data and information in an open dataset, given that indices for some gauges cannot be shared directly due to data licence restrictions. The motivation for gridded indices within GSDR-I is thus similar to that for the GHCNDEX and the HadEX family of gridded indices. These datasets also guided the order of operations for gridding in GSDR-I. Specifically, indices were first calculated for each gauge record and then the resulting indices were gridded (i.e. index values were gridded, not anomalies).

This processing sequence remains the current standard for indices. As precipitation is highly variable in space and time but gauge networks are often sparse, it can be difficult to grid the gauge observations themselves in a satisfactory way^30^, whereas indices tend to be better correlated over larger distances^7^. Nevertheless, this sequence can lead to potentially significant differences in gridded index values compared with the reverse approach of gridding gauge data first and then calculating indices^1,31^. Therefore care is required in comparison with indices calculated from other data types (e.g. reanalyses, climate models or satellite)^32^ that tend to represent grid cell averages, as highlighted in the Usage Notes.

For comparability with GHCNDEX and the HadEX datasets, a modified angular distance weighting (ADW)^7,33^ method was employed for gridding. ADW has been shown to exhibit both good performance for gridding indices and robustness to parametric and structural uncertainties^34^, especially for precipitation^32^. The ADW method estimates index values at the centre of each grid cell using a weighted combination of values from surrounding gauges. Weights for the selected gauges are based first on their distances from the cell centre. The distance-based component of the weights are then scaled by a direction-dependent term, which is proportional to the bearing of each gauge from the cell centre relative to the bearings of all other selected gauges. The method is thus designed so that gauges gain higher weights if they are close to the grid cell centre and if they are relatively isolated rather than close to multiple other gauges. Full equations for the weights are provided in Appendix A1 in Dunn *et al*.^7^.

The indices were gridded on a regular 1° latitude-longitude grid. Gridding was undertaken using gauges with more than 80% completeness for the relevant period, i.e. the month/season/year for time series indices or the record period for supplementary statistics. A value was estimated at a grid cell if any stations with valid index values were available within the search radius explained below. Cell-wise metadata is provided on the number of gauges used in the gridding calculation (along with the number of gauges in each individual cell) for each field generated, which gives a user the option to set a higher threshold. Further details of the metadata are given below in the Data Records section. The gridding calculations were implemented using Python (see Code Availability section).

### Gridding: Search radius

For a given index and grid cell, gauges are selected for inclusion in ADW gridding using a search radius, which is determined from the rate of decay in correlations between pairs of stations as the separation distance between stations increases. The search radius is taken as the decorrelation length scale (DLS) or e-folding distance of this relationship. The DLS is estimated by binning the correlations for station pairs and fitting a relationship of the form $R=A{e}^{-x/b}+c$ to the bin-wise median correlation, where *R* is correlation, *x* is separation distance, and *A*, *b* and *c* are parameters to be estimated. Variable bin widths were used to resolve the relationship more accurately at relatively short separation distances. The first bin used was 50 km wide and the final bins were 500 km wide. The DLS is defined as the distance at which correlation drops to *A/e*. Only gauges with record lengths of 15 years or more were used to help identify the DLS and a maximum of 300 stations were used from each country for computational tractability.

For the time series indices, the DLS is calculated separately for each index and on a monthly, seasonal and annual basis (e.g. one DLS for all Januarys). It is also calculated separately for different latitudinal bands, which helps to account for the strong influence of latitude on precipitation climatology. The specific latitudinal bands overlap a little and differ from those used in HadEX3^7^, in order to account for the lesser availability and specific distribution of sub-daily precipitation gauge records. The bands used are: (1) 65°S to 25°S, (2) 30°S to 30°N and (3) 25°N to 65°N. The resulting DLS values were linearly interpolated to the latitudes of the grid cell centres to reduce boundary effects between latitudinal zones. Below 65°S and above 65°N the DLS of the closest mid-latitude band was used, given the very low gauge density at high latitudes.

Reflecting the potential for low spatial correlation of short duration precipitation and extremes, minimum and maximum DLS values are set at 100 km and 500 km, respectively. If insufficient data are available to calculate the DLS, a default value of 100 km is used for the time series indices. These values were determined after analysing the DLS calculations and testing a small number of alternatives. During gridding, if only one gauge was available within the search radius, an additional criterion was added to check whether this value was within 100 km of the cell centre. The supplementary statistics all use a DLS of 200 km in gridding, as correlations cannot be calculated because the statistics are not time-variant. The larger value of 200 km was selected after initial testing and reflects the higher spatial correlation of climatological quantities (e.g. percentiles) compared with index values for particular months, seasons or years. This testing also supported previous findings that the gauge density is a more significant factor than the DLS in interpolation or gridding accuracy^34,35^.

## Data Records

The data records presented in the GSDR-I dataset fall into two groups – gauge and gridded records. The first (gauge) group contains metadata, time series indices and supplementary statistics for each individual gauge. These data records are provided for 80% (14,832) of the total 18,591 gauges available to the study (i.e. with the total gauges being those used from GSDR plus the indices and statistics shared by partner organisations). Unfortunately, the indices and statistics cannot be released for all of the individual gauges, due to licensing restrictions. Of the 14,832 gauges for which indices are provided, 27% (4,008 gauges) are in Europe (primarily western Europe and Scandinavia) and 58% (8,614 gauges) are in North or Central America (including 6,813 in the United States and 1,801 in Canada). The remaining gauges with open indices are in Japan (1,757 gauges) and New Zealand (453 gauges).

Table 3 summarises the information available for the first (gauge) group of data records, i.e. the metadata, indices and statistics available for individual gauges. These data records lend themselves to a tabular structure and are published in.csv file format. Metadata for each gauge include information such as a gauge identifier, coordinates, elevation, record period and record completeness. The indices and statistics files contain basic coordinate metadata for convenience, alongside the index/statistic values and data completeness information. As noted above, data completeness for time series indices indicates the completeness of the underlying data series for a given month, season or year. For supplementary statistics, data completeness indicates the threshold used for inclusion of a given month, season or year in calculations, with the number of valid data points also provided. To keep individual file sizes manageable, there are separate files for each aggregation period (monthly, seasonal or annual).

**Table 3:** Overview of table fields (columns) in each of the data records available for individual gauges in GSDR-I.

Data Record	Table Fields (Columns)
Gauge metadata	Gauge ID, country, latitude, longitude, elevation, record start date/time, record end date/time, time zone (UTC offset), percent missing, measurement resolution/precision
Time series indices (e.g. maxima)	Gauge ID, latitude, longitude, year, (season), (month), index value, percent completeness
Supplementary statistics (extreme percentiles and diurnal cycle/profile)	Gauge ID, latitude, longitude, (season), (month), (hour), record completeness threshold, number of years meeting completeness threshold, value of statistic

Brackets indicate which fields are present only if relevant, e.g. monthly Rx1hr will include a month column. For the diurnal cycle supplementary statistics, the value for each hour of the day is provided in its own column and an additional column records the number of wet days for the period.

The second group of records in GSDR-I is the gridded indices and statistics, which bring in information from all available gauges. The gridded data records are published as Network Common Data Form (NetCDF) files following the NetCDF Climate and Forecast (CF) Metadata Convention (CF1-8). These files are the modern standard for many spatiotemporal climate datasets, such as climate model outputs and meteorological reanalyses, as well as other gridded indices datasets like HadEX3 and GHCNDEX. NetCDF files are platform-independent and self-describing, with each file containing headers and metadata as key/value attributes. In addition to the gridded indices and statistics themselves, the files contain spatial fields with additional metadata (see Table 4), including the number of gauges used in gridding calculations at each grid cell. All of the indices listed in Tables 1, 2 are provided in the gridded data record on a monthly, seasonal and annual basis.

**Table 4:** Overview of key variables in NetCDF files of gridded indices (in addition to standard variables of time, latitude and longitude).

Variable	Description
index (e.g. Rx1hr)	Index (e.g. Rx1hr) or statistic (e.g. percentile) value obtained via angular distance weighting gridding
n_cell	Number of gauges available within grid cell for calculation
n_radius	Number of gauges within search radius of grid cell centre (i.e. total number of gauges used in calculation)
cell_statistics	Index value for grid cell obtained by summarising gauges within grid cell (i.e. providing minimum, median, mean and maximum of index/statistic values for those gauges within the grid cell)
n_years	For supplementary statistics (percentile, diurnal profile) only, mean number of years of record available from gauges contributing to the gridded values

Both the gauge and gridded components of the GSDR-I dataset are available in one data repository^36^: 10.5281/zenodo.7492812.

## Technical Validation

There are three aspects to the technical validation of the GSDR-I dataset: (1) verification of the quality control procedure applied to gauge records, (2) validation of the indices calculations, and (3) assessment of the gridding methodology. Lewis *et al*.^23^ considered the first aspect in detail, finding that the GSDR-QC procedure improved correspondence between GSDR gauges and high-quality daily reference gauge series archived by the GPCC. Lewis *et al*. also evaluated the GSDR-QC procedure against a sample of 300 manually-checked GSDR gauges. The GSDR-QC procedure showed over 99% agreement with manual judgments of hourly data quality. More specifically, the false positive rate (i.e. incorrect removal of reasonable values) was low at 0.4%, while the true positive rate (i.e. correct removal of erroneous values) was 71%. There are inevitable trade-offs in automated QC procedures, but GSDR-QC achieves a good balance between removing erroneous data and retaining reasonable data, with prioritisation of a low false positive rate to avoid unduly removing important extremes^37^.

**Fig. 3:**
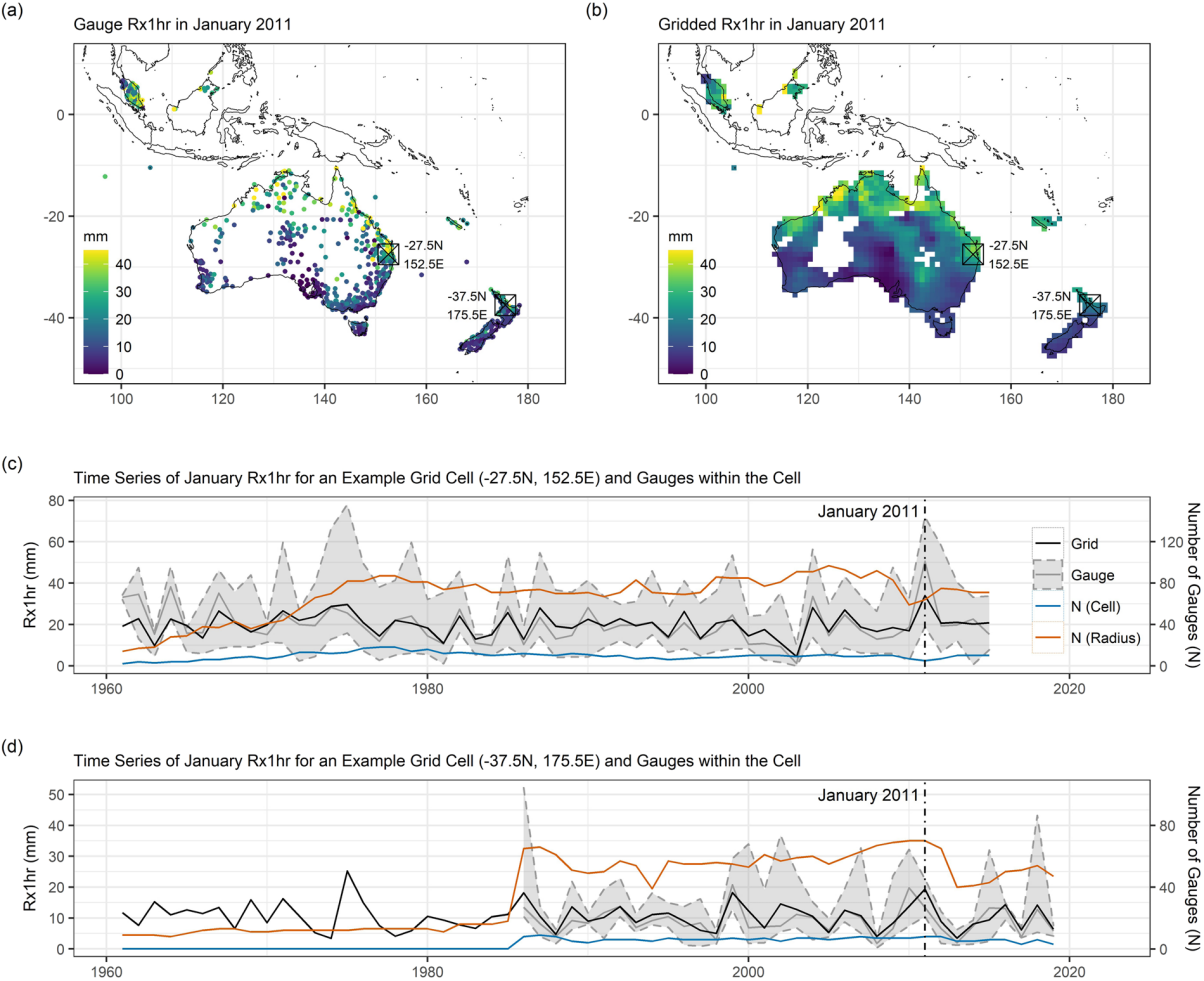
Comparison of Rx1hr index from gauge records and gridding. One example month (January 2011) is shown in (**a**) for Rx1hr at each available gauge (>80% completeness), with the equivalent gridded field given in (**b**). Time series of Rx1hr (January values only) for two example grid cells are shown in (**c**) and (**d**), along with the median (line) and range (shading) of Rx1hr values from gauges within the grid cells. The number of gauges (N) available in the grid cell (N (Cell)) and in the search radius used in gridding (N (Radius)) are additionally plotted.

Validation of the indices calculations was achieved by selecting a sample of gauges and checking each of the calculations/outputs manually in detail. After calculation of the indices for all gauges, preliminary maps and time series plots were also generated to check for outliers and ensure that the spatial/temporal patterns were coherent with conceptual understanding of spatiotemporal climatic variation. These plots are available in a repository^38^ at 10.5281/zenodo.7492848 (see also examples in Fig. 3). The majority of the indices build on simple and common concepts (e.g. maxima, counts of threshold exceedances, etc.), such that the software and methodological complexity of indices calculations is generally lower than for both the quality control and gridding procedures.

The gridded indices were evaluated first by qualitative comparison of maps of gauge indices with gridded indices for a selection of different examples. This comparison provides an overall sense check on the gridding, as the fields should show good qualitative consistency with the gauge values. One example is given in Fig. 3, which shows clear consistency in maps of gauge (Fig. 3a) and gridded (Fig. 3b) 1-hour precipitation maxima (Rx1hr) for a selected month (January 2011) in the South-East Asia and Oceania region. Selecting two example grid cells, Fig. 3c shows a good degree of correspondence between the time series for the grid cells and the time series for (the median of) the gauges present within the grid cells. Note that a perfect match is not expected here, in part because the gridding procedure draws additionally on gauges outside of the grid cell to produce its estimates. Coherence was also found in the other examples checked and available in the validation figures repository^38^.

Following previous evaluations of the angular distance weighting method^35^, further validation of the gridded indices was undertaken through cross-validation. Each gauge was left out of the indices dataset in turn and the angular distance weighting method was used to estimate the index values at the gauge location. Differences (residuals) between the estimated and observed index values provide a measure of the accuracy and uncertainty of the interpolation method. The cross-validation procedure was repeated for each index and each month, season and year using all available gauge index values with less than 20% missing data in the underlying records. The period 2001 to 2010 was used for cross-validation of times series indices. As noted in prior studies^35^, angular distance weighting is not an exact interpolator, which should be borne in mind when interpreting the residuals. However, cross-validation is still expected to provide a strong indication of the performance of the method, particularly in terms of bias and relative variations in skill according to season and location.

**Fig. 4:**
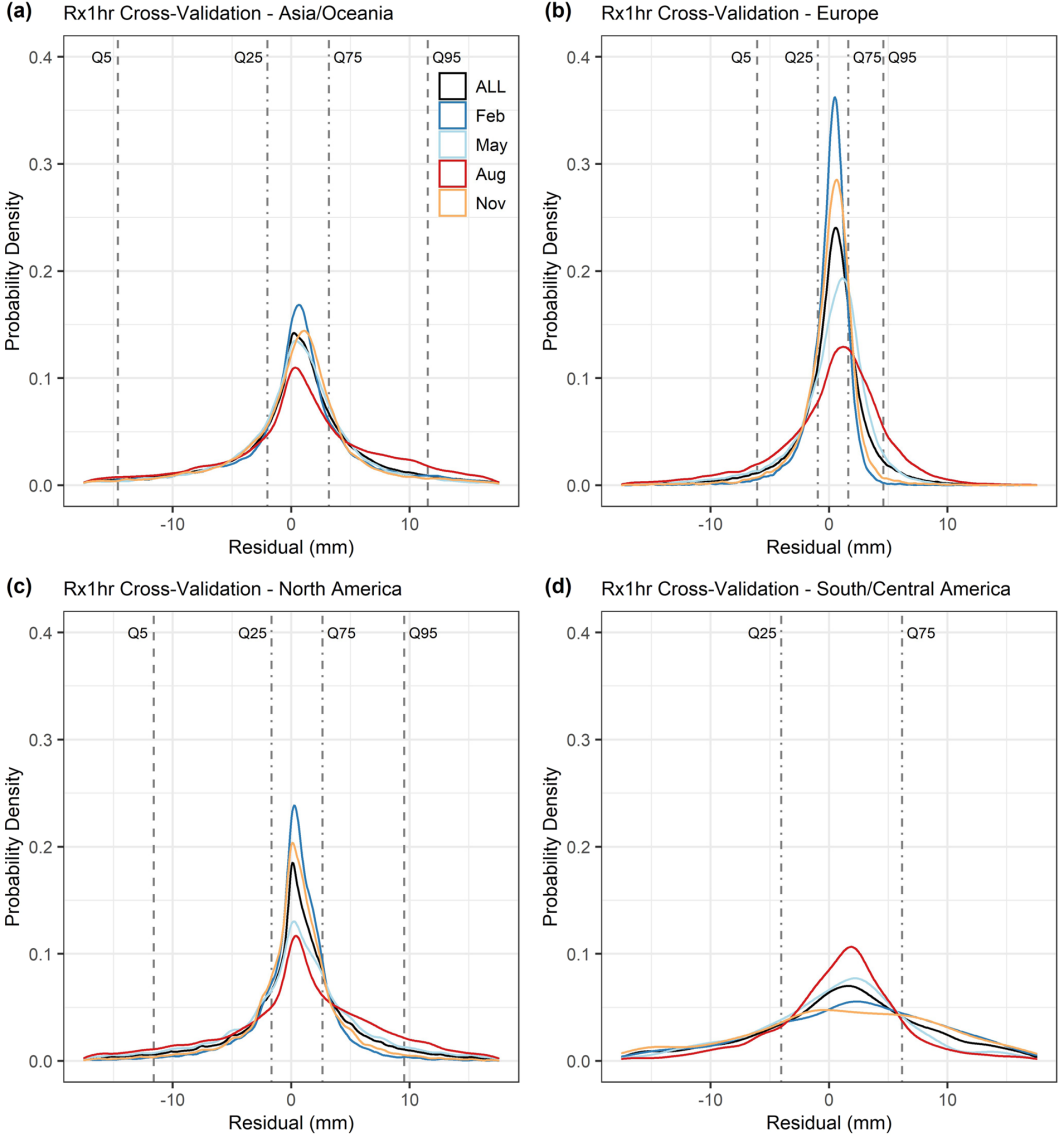
Distribution of residuals determined through eave-one-out cross-validation using monthly Rx1hr index values (2001 to 2010 period) at gauge locations. Residuals are shown for all months pooled together (ALL), as well as four example months. Each panel shows the distribution of residuals for a different region: (**a**) Asia/Oceania, (**b)** Europe, (**c**) North America and (**d**) South/Central America. Dashed lines indicate the 5^th^, 25^th^, 75^th^ and 95^th^ percentiles of the distribution of residuals (from all months pooled together).

The distribution of residuals for one example index (monthly Rx1hr) for four major regions (see Fig. 2) is shown in Fig. 4. Distributions are provided for all months pooled together, as well as separately for four example months. In all cases the distributions of residuals peak close to zero, which indicates that the gridding method does not exhibit large overall biases. The interquartile range of the residuals is mostly small at around 5 mm or less. However, Fig. 4 also shows notable spatial and seasonal variation in the degree of spread in the residuals. In most regions, the distribution of residuals is peakiest in February and most widely dispersed in August. The contrast likely reflects seasonal variation in the balance between different types of precipitation (e.g. stratiform and convective – with the former typically exhibiting much larger correlation length scales than the latter).

Spatially, the European region shows the tightest concentration of residuals around zero in Fig. 4, especially in winter. This suggests the gridding method is likely to work particularly well here. Performance for the North America region is likely to be a little better than in Asia/Oceania, whereas the more data-sparse South/Central America region shows the largest dispersion. In all regions it is noticeable that, although most residuals are relatively small, there are some notably larger ones present (i.e. in the tails of the distributions). These large residuals are often associated with areas of sparse gauge density or complex topography, which provide longstanding challenges when attempting to derive representative index values^32^. That said, other example plots of residuals determined through cross-validation (available in the validation figures repository^38^) confirm that overall the gridding method works well for different indices.

## Usage Notes

Specific points to bear in mind when using the GSDR-I dataset are:

1. The GSDR-QC quality control procedure focuses on errors arising from instrumentation, transmission or recording problems. It does not deal with issues affecting gauge representativeness (e.g. gauge type or siting) or systematic biases, such as those arising from wind-induced undercatch. This is not a shortcoming specific to GSDR-I; other precipitation indices and datasets are also typically unadjusted for these issues (including HadEX and GHCNDEX gridded ETCCDI indices). However, it is a point of uncertainty to bear in mind when analysing the dataset.
2. Data records for the supplementary statistics are based on the available record periods for each gauge (including only months, seasons or years when the data completeness threshold is met). This means that the record periods vary between gauges. It is possible for the user to calculate summaries of time series indices to focus on specific reference periods, e.g. 1961–1990. Depending on the region under consideration, this may rule out a substantial amount of data.
3. Gridding in the GSDR-I dataset proceeds by first calculating indices based on gauge time series and then gridding the resulting indices. The “order of operations” is thus the same as for the gridded GHCNDEX and HadEX family of ETCCDI indices. However, the reverse order (first gridding precipitation observations and then calculating indices from the gridded fields) is considered to be more directly comparable with climate model outputs, as well as other datasets in which grid cells represent spatial averages (e.g. meteorological reanalyses, satellite precipitation datasets, etc.)^1^. The difference between the two approaches can vary depending on region, season and the quantity of interest (e.g. absolute values compared with an indication of variability or anomalies), but it may be significant^31^. It is therefore important to consider this point when using GSDR-I in comparisons with other data products or model outputs.
4. The gridding method is likely to give a generally representative value, but it is not an explicit grid cell average. This consideration is additional to the order of operations point above, as the grid cell value may exhibit lesser representativeness in areas of complex terrain or highly variable precipitation patterns, e.g. places or seasons dominated by convective rainfall. Further research could also explore different indices gridding methods in more detail^7,32^, but this is a large task beyond the scope of this work.
5. Great care is required for any change or trend analysis undertaken with GSDR-I time series. Sub-daily precipitation records are typically much shorter than daily precipitation records, such that many of the gauges within GSDR are intrinsically unsuitable for climate change detection at present. It should also be noted that homogeneity checks are conducted on gauge time series with GSDR-QC, but these are not currently used to remove inhomogeneous records or to attempt to homogenise time series. Users should consult the available metadata before performing analyses sensitive to homogeneity issues. In general, the GSDR-I data records are better suited to characterising the spatial, seasonal and inter-annual variation in sub-daily precipitation variability and extremes at present, until such time as long records become available for more than a small number of countries.
6. In addition, the gridded indices use all available data to provide the best estimate for each month, season or year at a grid cell, so it should be remembered that the number of gauges incorporated often varies through time. This may introduce inhomogeneity into time series in the gridded dataset. Again, the gauge and gridded indices may therefore provide a better guide to variability rather than long-term changes – at least at this stage in the development of sub-daily indices. Further updates to the dataset over time will address this point by extending record lengths and opening up the possibility of producing a more homogeneous version of GSDR-I to support climate change detection work.
7. It is hoped that updates and extensions to the GSDR-I dataset will be carried out every few years, depending on resources, uptake and community contributions. As more observation data becomes openly accessible, these updates will ideally include the collation of additional gauge records that are not currently held in GSDR. This would help to fill in some of the gaps in spatial coverage across parts of Asia, Africa and South America. Over the longer term, it would be useful to automate the collection and processing of the data as far as possible, so that the GSDR-I indices dataset could be more frequently updated.

## Data Availability

The Python code for indices calculation based on gauge records and subsequent gridding is available in the code repository^39^ here: 10.5281/zenodo.7492877.
